# The Global Burden of Alveolar Echinococcosis

**DOI:** 10.1371/journal.pntd.0000722

**Published:** 2010-06-22

**Authors:** Paul R. Torgerson, Krista Keller, Mellissa Magnotta, Natalie Ragland

**Affiliations:** 1 Ross University School of Veterinary Medicine, St. Kitts, West Indies; 2 Section of Epidemiology, Vetsuisse Faculty, University of Zurich, Zurich, Switzerland; London School of Hygiene & Tropical Medicine, United Kingdom

## Abstract

**Background:**

Human alveolar echinococcosis (AE) is known to be common in certain rural communities in China whilst it is generally rare and sporadic elsewhere. The objective of this study was to provide a first estimate of the global incidence of this disease by country. The second objective was to estimate the global disease burden using age and gender stratified incidences and estimated life expectancy with the disease from previous results of survival analysis. Disability weights were suggested from previous burden studies on echinococcosis.

**Methodology/Principal Findings:**

We undertook a detailed review of published literature and data from other sources. We were unable to make a standardised systematic review as the quality of the data was highly variable from different countries and hence if we had used uniform inclusion criteria many endemic areas lacking data would not have been included. Therefore we used evidence based stochastic techniques to model uncertainty and other modelling and estimating techniques, particularly in regions where data quality was poor. We were able to make an estimate of the annual global incidence of disease and annual disease burden using standard techniques for calculation of DALYs. Our studies suggest that there are approximately 18,235 (CIs 11,900–28,200) new cases of AE per annum globally with 16,629 (91%) occurring in China and 1,606 outside China. Most of these cases are in regions where there is little treatment available and therefore will be fatal cases. Based on using disability weights for hepatic carcinoma and estimated age and gender specific incidence we were able to calculate that AE results in a median of 666,434 DALYs per annum (CIs 331,000-1.3 million).

**Conclusions/Significance:**

The global burden of AE is comparable to several diseases in the neglected tropical disease cluster and is likely to be one of the most important diseases in certain communities in rural China on the Tibetan plateau.

## Author Summary

Human alveolar echinococcosis (AE), caused by the larval stage of the fox tapeworm *Echinococcus multilocularis*, is amongst the world's most dangerous zoonoses. Transmission to humans is by consumption of parasite eggs which are excreted in the faeces of the definitive hosts: foxes and, increasingly, dogs. Transmission can be through contact with the definitive host or indirectly through contamination of food or possibly water with parasite eggs. We made an intensive search of English, Russian, Chinese and other language databases. We targeted data which could give country specific incidence or prevalence of disease and searched for data from every country we believed to be endemic for AE. We also used data from other sources (often unpublished). From this information we were able to make an estimate of the annual global incidence of disease and disease burden using standard techniques for calculation of DALYs. Our studies suggest that AE results in a median of 18,235 cases globally with a burden of 666,433 DALYs per annum. This is the first estimate of the global burden of AE both in terms of global incidence and DALYs and demonstrates the burden of AE is comparable to several diseases in the neglected tropical disease cluster.

## Introduction

**Figure 1:**
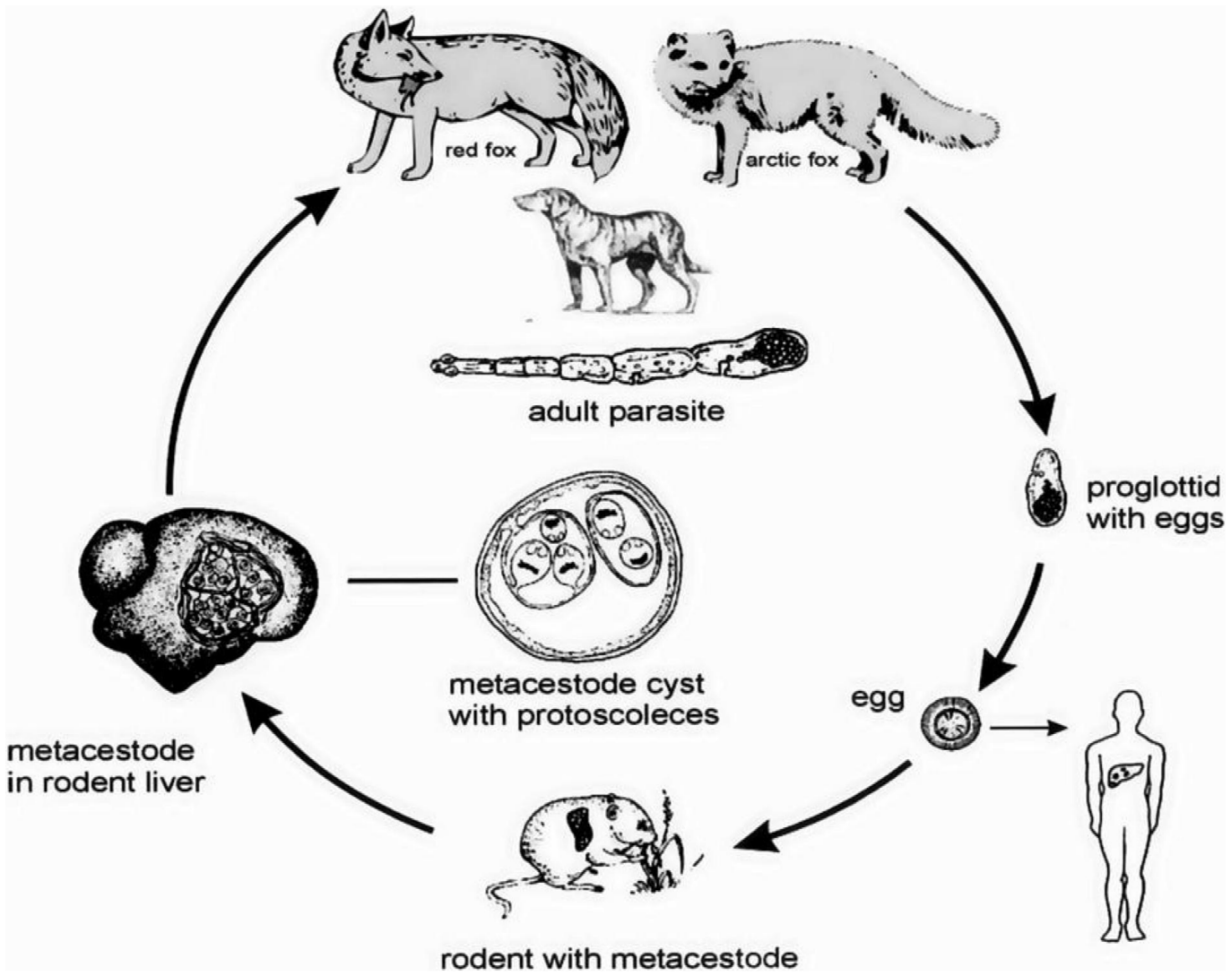
The life cycle of *Echinococcus multilocularis*. Man is infected as an aberrant intermediate host.

**Figure 2:**
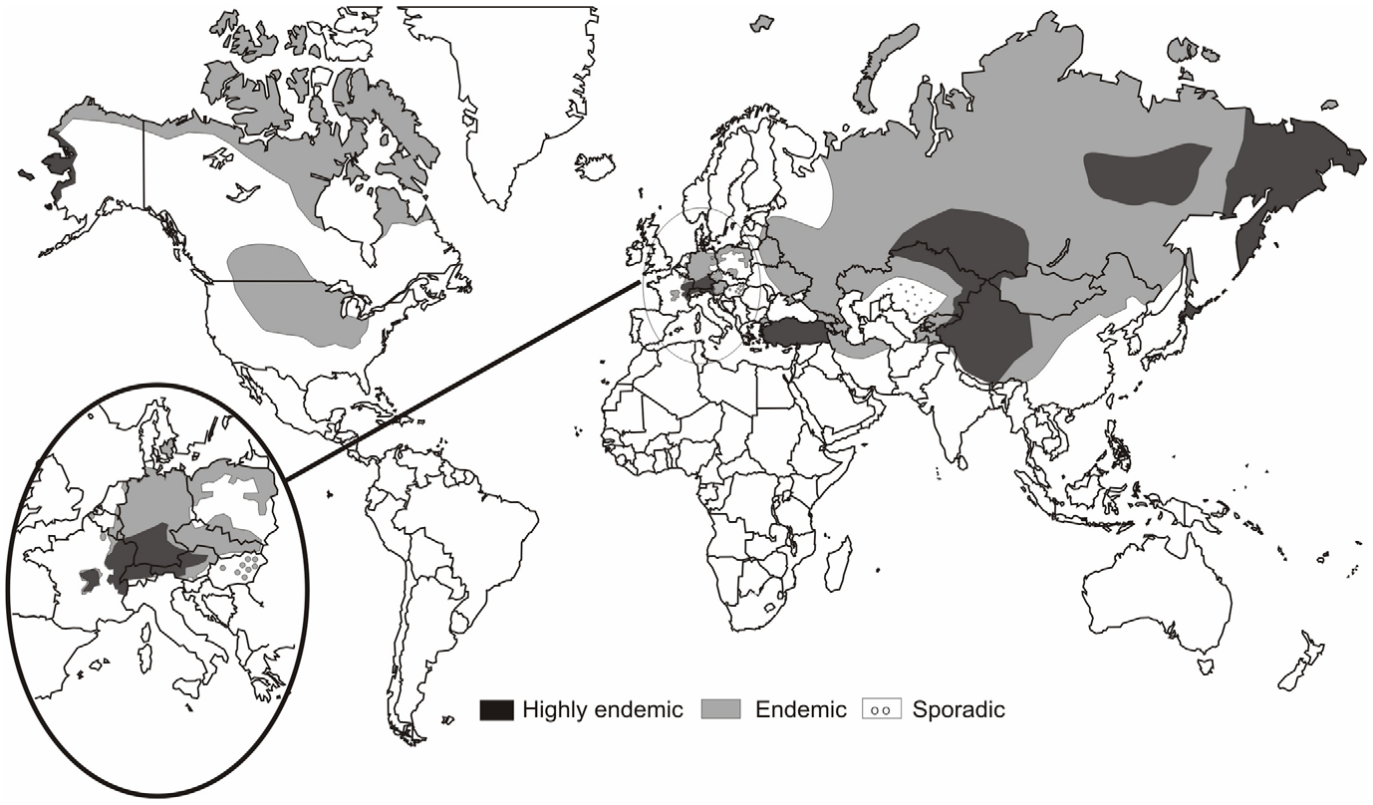
Global distribution of AE.

Human alveolar echinococcosis (AE) is caused by the larval stage of the fox tapeworm *Echinococcus multilocularis*. It is amongst the world's most dangerous zoonoses. Transmission of AE to humans is by consumption of parasite eggs which are excreted in the faeces of the definitive hosts: foxes and, increasingly, dogs. Naturally the parasite transmits between foxes or dogs and small mammals whilst humans are aberrant intermediate hosts (Figure 1). Human infection can be through direct contact with the definitive host or indirectly through contamination of food or possibly water with parasite eggs. Geographically *E. multilocularis* is confined to the northern hemisphere, but within that range has a wide distribution (Figure 2) [1]. In humans, infection results in a metacestode in the liver. This is a slowly growing infiltrative space occupying lesion. If untreated, this lesion will result in clinical signs such as abdominal mass and/or pain, jaundice, and ultimately liver failure [2]. In the late stages of the disease, the parasitic lesion can metastasize resulting in a variety of symptoms. Treatment options include liver resection to remove the parasite mass and chemotherapy using benzimadazoles is now being increasingly used. Survival analysis indicates that with judicious surgical and chemotherapeutic treatment the prognosis is relatively good [3]. However, chemotherapy is required continuously for many years, sometimes for the remainder of the patient's life to achieve success. In the absence of this expensive treatment the disease normally has a fatal course.

Human AE is an emerging disease in Europe. Studies in wildlife are detecting the parasite in new areas [4]. It is not yet clear if the parasite range is spreading or increasing surveillance has lead to greater detection rates. What is certain is that increasing fox populations in Europe are correlated with the greater numbers of cases of AE reported in Switzerland [5]. In Asia a major endemic focus was detected in China [6] with large numbers of human cases – in some communities 5% or more of the population is infected [7]. In such areas there is, not only high infection rates in humans, but a high prevalence of infection with the adult parasite in the dog population [8]. In central Asia there are also reports of a spill over of *E. multilocularis* into the dog population [9] and this may indicate an increasing threat of transmission to humans. Elsewhere there are increasing reports of AE being detected by surgeons in countries such as Russia and Turkey.

Control of the parasite is possible, for example, through the judicious use of praziquantel baits distributed to foxes [10]. Some studies have indicated that risk factors such as contamination of food or water are a likely conduit of human infection [11], [12] and could suggest alternative strategies to prevent human infection. However, whatever the intervention strategy, the economic efficiency of control will depend upon the societal burden of disease. The purpose of this study was to estimate the annual global burden of AE.

## Materials and Methods

Initially all countries, endemic for *E. multilocularis*, were identified. These were countries that were known to have autochthonous human cases of AE and/or *E. multilocularis* identified in animal populations. In addition, neighbouring countries where there were no known reports were also identified as likely endemic areas. The list of countries believed to be endemic for *E. multilocularis* is given in Table 1.

**Table 1:** Countries believed to be endemic for *E. multilocularis* over at least part of their territory.

Europe	Asia	North America
Austria	Afghanistan	Canada
Belgium	Armenia	USA
Belorussia	Azerbaijan	
Bulgaria	Bhutan	
Croatia	China	
Czech Republic	Georgia	
Denmark	India	
Estonia	Iran	
France	Iraq	
FYR Macedonia	Japan	
Germany	Kazakhstan	
Greece	Kyrgyzstan	
Hungary	Nepal	
Italy	Mongolia	
Kosovo	Pakistan	
Latvia	Russia*	
**Liechtenstein**	Tajikistan	
Lithuania	Turkey	
Luxembourg	Turkmenistan	
Moldova	Uzbekistan	
Montenegro		
Netherlands		
Poland		
Rumania		
Russia*		
Serbia		
Slovakia		
Slovenia		
Switzerland		
Ukraine		

*Both European and Asian parts of Russia include large endemic areas.

### Sources of information

Literature searches were undertaken in any relevant databases that could be accessed. These included all the following scientific databases: Pubmed, Medline, Science Citation Index, Scopus, East View (Chinese and Russian databases), Russian Scientific electronic library, and Google Scholar. This was supplemented by direct contacts with known persons working in the field in various countries. In addition, further information was solicited by directly contacting individuals who had authored manuscripts by the email address in the correspondence section. Key words used were *Echinococcus multilocularis* and alveolar echinococcosis for initial screening. Where appropriate the search term was also translated into the language of the relevant database. For each country a systematic search was undertaken to locate data from that country eg *Echinococcus multilocularis* AND France. All literature was initially screened. Most literature was not useful for calculating incidence rates (for example individual surgical case reports); although in a number of cases such individual reports confirmed that the disease was endemic in the country or region of interest. Inclusion criteria depended on the amount of available information from that country. Because of wide variability in the quality of the data from different countries it was not possible to use a standard procedure across all endemic countries. When there were extensive prevalence and/or incidence reports, particularly indicating whole country incidences, these were used as the primary data sources. However, for many if not most countries, such data was not available. In these cases the reports of individual cases or case studies were utilized to, at the very least, prove the presence of the disease (and in a few cases there were only reports from animal infections). The body of literature on experimental research (eg experimental infections of definitive or intermediate hosts) was, in the whole, of little relevance to this study.

### Calculations of incidence

For a number of countries such as Switzerland [5] or Germany [13], accurate figures for the annual numbers of cases were easily identified. This is because they had up to date national databases and/or accurate methodology for capturing the estimated numbers of cases each year. In other countries, such as Kyrgyzstan, accurate reporting figures were available based on histological confirmed cases presented for treatment from hospitals (unpublished). However, it is believed that, in low-income countries such as Kyrgyzstan, the reported cases are likely to substantially underestimate the total numbers of cases as a substantive number of cases are likely to remain undiagnosed because of the relative expense of seeking medical treatment.

China is believed to account for the majority of global AE cases. Estimates were based on mass screenings by ultrasound giving a prevalence estimate. Many such reports also indicated groups (such as Tibetan pastoralists) who were at particular risk of infection. In this region there were a number of large prevalence studies and epidemiological studies based on ultrasound confirmation of diagnosis. These often consisted of several thousand individuals and hence gave samples of populations at risk. In addition particular groups at risk such as Tibetan pastoralists were identified. The studies used are given in Table 2. In total in studies spanning the first decade of the 21^st^ century over 36,000 individuals have been screen by ultrasound over large areas of Ningxia autonomous region, Sichuan, Gansu, and Qinghai provinces. The total number of individuals currently affected with AE was estimated from these prevalence studies and the total populations at risk. This data was then converted into an annual incidence of AE for these populations. Survival analysis of a series of cases from Switzerland suggested that the 50% survival rate of approximately 8 years if treatment is not available for individuals with a mean age of presentation in their early 50s [3]. This can rise to approximately 11 years for younger subjects. If endemic stability is assumed then there will be approximately 6.1% of infected young adults dying from the disease rising to approximately 8.2% of infected adults who are aged in their early 50s. Hence the annual incidence is approximate 6.1%–8.2% of the detected ultrasound prevalence in the population of these respected age groups.

**Table 2:** Population studies for AE in rural China.

Region	Number with AE	Population size studied	Reference
Ningxia (Xija, Guyuan and Haiyuan counties	96	4778	[51]
Sichuan (Ganzu autonomous prefecture)	308	8512	[52]
Gansu (Dingxi prefecture)	114	3836	[53]
Qinghai	39	1549	[54]
Sichuan	60	705	[55]
Gansu	84	2482	[56]
Gansu (Ming and Zhang counties)	86	2485	[57]
Qinghai (Zhiduo County)	2	979	[58]
Qinghai (Chindu, Zeko and Gade counties)	31	3703	[59]
Sichuan (Ganzi and Shiqu counties)	223	7138	[12]
Ningxia Hui Autonomous Region (Xiji County)	20	221	[60]
*Total	1063	36388	2.9%
+Gansu (Zhang county)	65	1312	[6]

*Total of studies in the last 10 years.

+Study from 1992.

China has large populations in rural areas that are potentially exposed to this parasite. An estimate was performed of the population at risk by examining population data county by county from Chinese Census data. The estimates of the mean prevalence in the population at risk were estimated from relative risk of various ethnic communities from population studies and the proportions these communities make up in the general population. There are also reports of AE in inner Mongolia and Xinjiang but these tend to have considerably few cases then the main endmic area of the Tibetan plateau.This prevalence data was extrapolated to the estimated population at risk and then converted to incidence based on the results of survival analysis [3].

**Figure 3:**
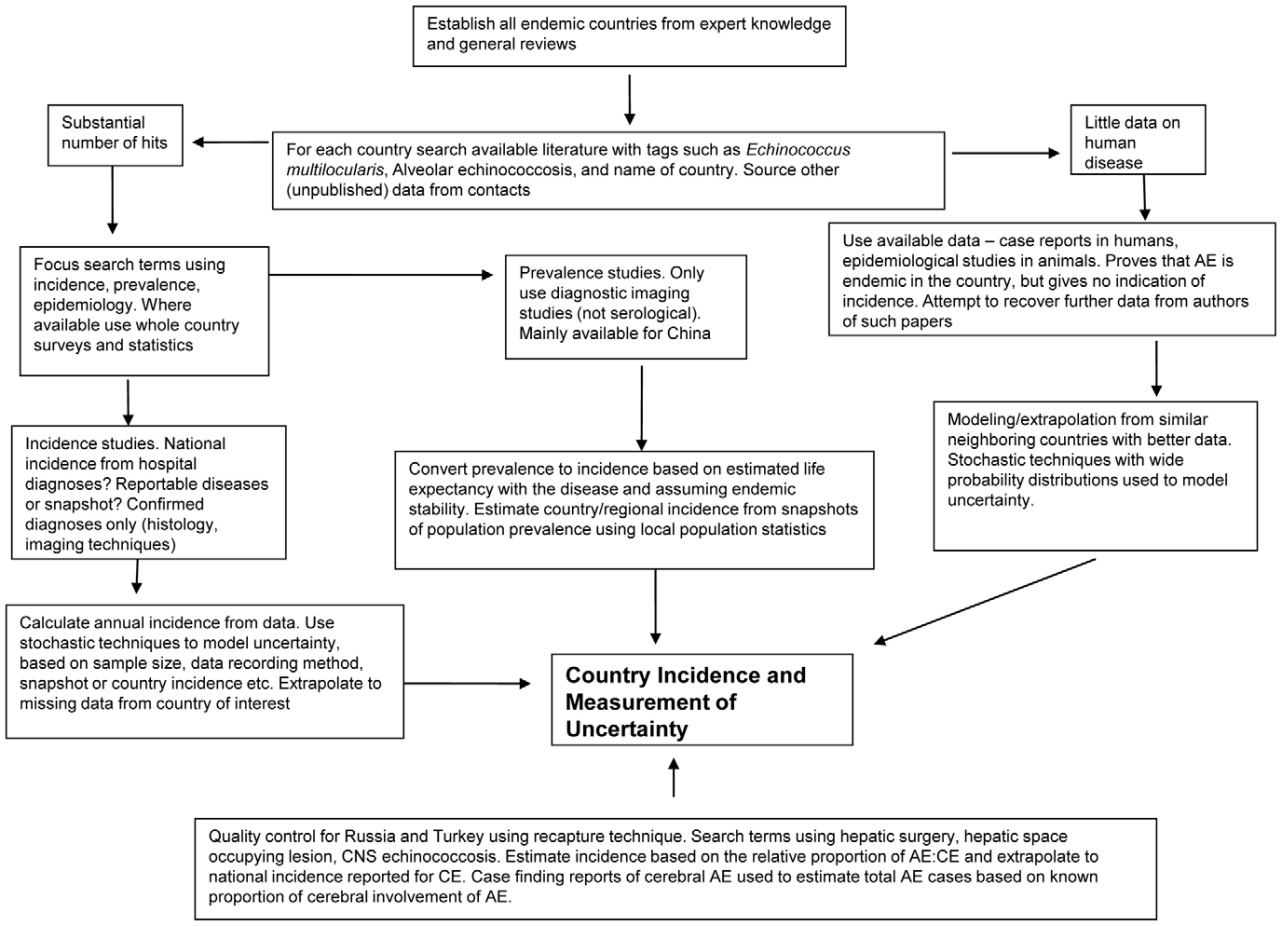
Flow chart illustrating the search methods and processing of information.

A flow chart illustrating the methodology used in the study is given in figure 3.

### Incidence estimates from case series

In Turkey and Russia total numbers of cases for echinococcosis are recorded. In Russia in 2002 there were 3,274 cases of cystic echinococcosis (CE) notified [14]. In Turkey 14,789 cases of CE were notified in the 5 years 2001–2005 [15]. Separate information for AE was not available. This may be because it has only recently been made notifiable such as in Turkey [16] or because CE and AE cases are not distinguished in official figures. Despite this, in Turkey there are some nationwide figures which give a minimum estimate. However, there are a number of case series of echinococcosis published by surgical units which differentiate between CE and AE. The relative proportion of AE to CE cases in such surveys can be used to estimate the likely number of AE cases for the whole country. These case series (Tables 3 and 4) are a means to calculate the total incidence of AE from the relative incidence and total country incidence of CE. In addition, in Turkey there was one detailed case series of echinococcosis with CNS involvement that identified 16 cases of AE with cerebral involvement over a 5 year period from neurosurgical units in Turkey [17]. A large study in China suggested that 4% of AE cases had neurological involvement [18]. Likewise, a large European study found 17 of 559 (3%) cases of AE had brain involvement [19]. The likely number of AE cases can then be estimated by assuming that the proportion of cerebral AE cases in Turkey was similar to these reported case series.

**Table 3:** The relative numbers of CE∶AE cases in various studies from Turkey.

Number of CE	Number of AE	Reference
44	6	[61]
72	8	[62]
196	47	[63]
109	39	[64]
203	16	[17] *
336	11	[17] **
138	20	[65]
111	22	[66]

*Intracranial echinococcosis only. Case searching from 47 neurosurgical units between 1994 and 1999.

**Intracranial echinococcosis only. Reported literature cases from Turkey from 1940s–1990s.

**Table 4:** Published case report series from Russia.

Number of CE	Number of AE	Reference
48	24	[67]
95	40	[68]
44	84	[27] *

*These data are from districts of Siberia where AE relative incidence might be expected to be higher.

### Estimates of DALYs

To calculate DALYs standard techniques were used [20]. The years of life lost (YLLs) were calculated on the assumption that the disease is fatal within an average of 8 years of diagnosis if untreated. If treatment is available then the prognosis was assumed to be reasonable with just 2–3 YLLs. These assumptions are based on previously published survival analysis [3]. In order to calculate the years lived with disability (YLDs) is was necessary to assign a disability weight. As no accepted disability weight has yet been assigned to alveolar echinococcosis the disability weight for carcinoma of the liver was used as previously [7]. The years lived with the disability again depends on where the cases are presented and available treatment options. Where there is no treatment, death can be assumed within a mean of 8 years for patients in their 50s, but this increases to 11 years for someone presenting in their 20s [3]. In low income countries a disability weight for pre terminal liver cancer (0.200) was assigned for 6–9 years with a disability weight for 2 years living at the disability weight for metastatic and terminal stages (0.75–0.81) [21] .The number of years at these weights depended on the age specific incidence (see below). In those countries where advanced medical treatments lead to a successful outcome, a disability weight of 0.200 for mild disease for the average length of treatment (7 years) [3]. This was based on the fact that liver cancer has similar symptomatology to AE [7].

### Age and gender specific incidence

This data are important in estimating the burden of disease. For Europe a large data set of 559 [19] was used as the basis for the age and gender distribution of cases and hence the age weighting and YLLs for the estimated DALYs. For China prevalence data only was available, but much was age and gender stratified and was used to calculate the age and gender specific incidence after adjusting for bias by comparing the sampled population with the age and gender profile of the general population using census data [7]. Other counties including Turkey had case series reports which indicated age and gender of cases, although in some instances only the mean age was given. Unpublished data for case series from Kyrgyzstan were used and assumed to be representative of similar ex-Soviet states where data was not available.

### Stochastic analysis

Based on the quality of the data we were able to assume some countries (eg Switzerland) had accurate estimates of incidence, whilst others it was much more uncertain, particularly when estimates had to be created from modelling or extrapolation from neighbouring regions. A Monte-Carlo routine was written to resample estimates of incidence from each country based on the likely probability distribution of the total case incidence. This was similar to methods described previously [7], [22]. Distributions were based on a number of factors from the available data including possibilities of missing data.

## Results

### Incidence

For China in total we estimated there are 230,000 individuals presently suffering from AE and a total population at risk of some 22.6 million in 7 provinces (Table 5). Assuming most of these go untreated and hence have a fatal outcome, this was used to estimate the incidence from age stratified life expectancy following diagnosis. This gives an annual incidence of approximately 16,629 new cases per annum.

**Table 5:** Estimated annual numbers of new cases in endemic provinces of China.

Chinese province	Population at risk	Estimated prevalence	Estimated median number of new cases per year
Gansu	3.6 million	2.9%	7676
Inner Mongolia	3 million	0.02%	44
Qinghai	5.4 million	1.0%	3766
Ningxia	1.2 million	2.0%	1770
Sichuan	0.92 million	3.6%	2390
Tibet Autonomous Region	2.7 million	0.1%	172
Xinjiang	5.8 million	0.2%	811

Russia is a huge endemic area stretching from Eastern Europe to Siberia. We estimated that there are approximately 1,180 cases per year in this country. AE is found throughout the northern parts of Asia with important foci in central Asia and Turkey. The estimates for the annual numbers of cases in Asia excluding Russia are given in Table 6. The estimated numbers of cases from Europe from countries that are endemic for AE are given in the Tables 7 and 8, with the references supporting the estimate. Although North America is endemic for *E. multilocularis* in animal hosts there is very little evidence for transmission to humans presently.

**Table 6:** Estimated annual incidence of AE in Asia, by country.

Country	Estimated Number of Cases	
Afghanistan	1	Single case report [31]
Armenia	3	Estimated
Azerbaijan	6	Estimated
Bhutan	<1	No data
China	16,629	See table 5
Georgia	6	Estimated
India	1	Two case reports [29], [30]
Iran	11	Estimated
Iraq	1	Single case report [33]
Japan	12	Reported cases [69]
Kazakhstan	39	Estimated
Kyrgyztan	17	Actual figures (unpublished)
Mongolia	9	Actual figures (unpublished)
Nepal	<1	No data
Pakistan	<1	No data
Russia*	1180	See text
Tajikistan	20	Estimated
Turkmenistan	2	Estimated
Turkey	100	Estimated and modelled from various data (see text)
Uzbekistan	24	Estimated

*Including European Russia.

**Table 7:** Estimated median annual numbers of cases from Eastern Europe.

Country	Estimated annual number of cases	Source
Belorussia	6	Border districts of Lithuania have cases
Bulgaria	1	Estimated
Czech republic	1	Estimated
Estonia	9	Similar to Lithuania
Greece	1	1 case reported from 1980 to 2000 [70]
Hungary	1	First case reported 2004 [71]
Latvia	9	Similar to Lithuania
Lithuania	9	Reported cases [40]
Macedonia	1	One case reported in 10 years [72]
Moldova	1	Estimated
Poland	3	1992–2007, 45 cases recorded [73]
Slovakia	4	Four cases in 2007 [74]
Slovenia	2	0.45 per 100,000 over 5 years [75]
Ukraine	10	Estimated. Endemic [25]

**Table 8:** Estimated annual numbers of cases of AE in Central and Western Europe.

Country	Estimated annual number of cases	Reference
Austria	7	[19], [76]
Belgium	1	[19], [77]
France	21	[68]
Germany	61	[78]
Switzerland	20	[3], [5]

We estimate that the median estimate of the total numbers of AE cases in the world is 18,235cases per year with 95% CIs of 11,932–28,156. Of these 91% of cases are believed to be in China with just 1,606 occurring outside China.

### Estimated DALYs

Globally YLLs due to AE was estimated at 616,897 (CIs 296,485 – 1.2 million). Again China had most of YLLs and as a proportion was even higher than the incidence as it was assumed that the majority of cases in China did not receive treatment. The age of onset was also younger then compared to Europe. Thus, whilst China had 91% of the global number of cases it is believed that it has 95% of the YLLs due to alveolar echinococcosis. The total number of DALYs per annum for the world is estimated at a median of 666,433 (CIs 331,539 – 1.3 million).

## Discussion

This report represents a first attempt to estimate the global burden of AE although the global geographical distribution of *E. multilocularis* has been reviewed previously (e.g. [2]). Throughout much of its geographical range AE is sporadic in humans. In high income countries such as Germany and Switzerland the numbers of cases were the actual number reported (Switzerland) [5]. Alternatively in Germanyreported figures were based on a reported capture recapture technique which modelled underreporting [13]. These are believed to be accurate reports of the numbers of cases. Reviews of published data also gave estimates for a number of other upper income countries.

For some lower income studies there was limited official data (unpublished) reporting the total number of cases presenting for treatment. However in such countries a major underestimation of the numbers of cases is possible as only relatively wealthy individuals can pay for medical care and the majority of cases may not present for treatment and hence go undiagnosed. A criticism of our approach is that we did not use consistent inclusion criteria for data in different countries. What we did use was the best available data and balanced this inconsistency by using a stochastic approach to model uncertainty. In the countries where we believed the data was accurate a very narrow probability distribution was chosen for the Monte-Carlo routine. In contrast where there was poor data, a very wide distribution was used to model this uncertainty. Hence, the median incidence and estimated DALYs together with the 95% confidence limits give a good estimate of the burden of AE.

In certain districts of China several studies have indicated a very high prevalence of AE through mass screening studies. Surveys have consistently shown a high prevalence of AE using ultrasound studies across Sichuan, Gansu, Qinghai, and Ninxia (Table 2). This confirms that there are large numbers of AE cases in China and these represent at least 91% of the global incidence. In some communities the prevalence of AE is similar to that of tuberculosis [23]. Because of the large population at risk and the consistent finding of high prevalences we believe the estimate of the number of cases in China is representative. However, the prevalence is not uniform with variations within these districts of between <1% to 12%. The calculations have tried to accommodate these variations in arriving at an overall incidence figure. In the Tibet Autonomous Region (TAR), the incidence could be much higher than the figures suggested. Tibetan communities in neighbouring districts of Sichuan for example have very high prevalences. The parasite is known to be endemic in TAR, but there is no published human surveillance data. We were only able to assume that prevalences in the eastern most part of TAR were similar to prevalences in neighbouring counties of Sichuan or Qinghai. It is possible, therefore, that the true incidence could be thousands rather than the hundreds that are suggested. There is a single case report of cerebral AE in a Tibetan monk who originated from Lhasa which is quite some distance west of the known highly endemic areas of Gansu, Qinghai, and Sichaun [24] which is evidence that the parasite has a greater range than our conservative assumption. In Xingjiang the parasite is endemic, but human studies have mainly uncovered cases along the northwest of the province and hence the population at risk and actual case numbers are calculated accordingly.

Russia is a large endemic area for alveolar echinococcosis. In some districts, particularly in Siberia there are reports of a number of human cases whilst elsewhere the disease is sporadic. Detailed data is somewhat lacking despite intensive search of English and Russian language databases. Most data is reported in terms of hospital reports and is therefore estimated as an annual incidence. There are no mass ultrasound surveillance studies as in China although there are a few mass serological studies which tend to confirm the potential for large numbers of cases, especially in Siberia. It is clear, however, that the disease occurs sporadically across almost the entire country. Even in districts where there are no reported human cases there are reports of the parasite in animal hosts so transmission to humans is likely. The estimates for the whole of Russia initially relied on extrapolating data from districts where there were known reports or unpublished data. Bassonov wrote an extensive monograph [25] detailing the epidemiology of echinococcosis throughout countries of the former Soviet Union including summaries of otherwise difficult to obtain material. This was also used as a basis for estimating the numbers of cases in Russia. In addition, we were able to access some local Russian language reports including articles in local newspapers and these largely confirmed our assumptions [26]. Estimates based on the samples from case series (essentially a type of capture recapture technique) described in the text tended to confirm these estimates. We assumed a maximum ratio of 1∶2 for AE∶CE. However, one study of surgical cases from Omsk in western Siberia described 84 cases of AE and 44 cases of CE [27]. As most of these cases were from Siberian districts such as Kemerova, Altai, Yakutia and Tomsk which could indicate higher numbers of AE than the ratios we used in our calculations.

The mountainous regions of Kazakhstan, Kyrgystan, Uzbekistan, and Tadjikistan are all endemic for *E. multilocularis* [28]. Here the cycle of infection has been well described in terms of the animal hosts. The most accurate figures available are from Kyrgyzstan where approximately 35 cases per years are now being reported in the hospitals (unpublished figures from the Government Epidemiological Surveillance Unit, Bishkek). This figure is likely to be accurate in terms of numbers being treated. However, it may underestimate the actual numbers of cases occurring as the country is poor and expensive medical treatment is not available to a large part of the population.

Although ethnically distinct, in terms of geography and economy, Tadjikistan is very similar to Kyrgyzstan. Therefore, as it is also endemic for *E. multilocularis*, similar numbers of cases per year could be expected as there is likely a similar population size at risk. Kazakhstan and Uzebkistan are much larger countries in area and population but as much of their territory is in low or non-endemic areas the proportion of population at risk is smaller, but absolute numbers are similar. Turkmenistan is likely to have few cases of AE, as much of the territory is arid desert which is inimical to transmission. Mongolia has a number of reported cases. From unpublished data there were 9 cases of AE in both 2006 and 2007.

India, Nepal, Bhutan and Pakistan border on endemic zones and may have a few cases. The disease has been reported in India (Kashmir) [29], [30]. For Afghanistan, data is not available. However there is a case report of a patient originating from Afghanistan who was treated in the UK (which is non endemic) [31]. Therefore, further cases are likely, especially in the north of the country. Iran is endemic for AE but there is little data. Between 1948 and 1993, 37 cases of AE were reported [32] or less than 1 case per year. In view of the fact that neighbouring Turkey is known to be highly endemic this is likely underreported. There is a single case report of AE from northern Iraq [33].

Turkey is highly endemic for echinococcosis. In total, approximately 3000 cases are recorded annually. The number of cases of AE is uncertain. The proportion of cases of echinococcosis that are AE have been reported in a number of case series (Table 3). Assuming a similar ratio of AE to CE nationwide as found in the case report series this would suggest as many as 500 cases of AE per year. However, such selected case series may overestimate the true incidence of AE and these data contrast somewhat with the report of 206 cases of AE in Turkey for the period 1980–2000 or just 10 cases per year [34]. AE has only recently been made reportable in Turkey [16] and so more accurate figures are unavailable. Also of interest is a case finding study which specifically searched through the records of 47 neurosurgical units for cases of cerebral echinococcosis. This study found a total of 219 cases of intracranial echinococcosis in the five year period 1994–1999 [17]. Of these 16 were AE and 2 were AE with no extra CNS involvement. Another report found 4 CNS cases of AE in just 27 months at one centre [35]. Intracranial AE is thought to be rare with CNS involvement usually a manifestation of metastases from a primary lesion. A large study in China suggested that 4% of AE cases had neurological involvement [18]. Likewise, a large European study found 17 of 559 (3%) cases of AE had brain involvement [19]. The incidence of diagnosed intracranial cases of AE therefore gives strong evidence there must be at least 100 cases of AE per year in Turkey. This type of approach may, nevertheless, substantially underestimate AE cases in resource poor endemic regions, as a smaller proportion of AE cases my receive hospital treatment then are actually present in the community. For example, in south Ningxi in China, CE represented 96% of hospital treated cases of echinococcosis, but ultrasound studies in the local community suggest that 56% of cases of echinococcosis are AE [36].

The USA and Canada are endemic for *E. multilocularis*. However there is very little transmission to man. There have been reports from Native American communities of high incidence rates in Alaska [37] but only locally and these have been eliminated by appropriate intervention programs. Otherwise, there have only been single cases reports in Minnesota [38] and Manitoba [39]. Thus there is very little evidence for autochthonous human cases presently in North America despite the active transmission in animal hosts.

Eastern Europe has highly variable data. The parasite is endemic in most of the former Soviet States. Lithuania has the best data and reports incidence rates [40] and it is likely that the other Baltic States have similar incidences due to similarities in culture, geography, and population. From central and western Europe there is usually high quality data giving details of the numbers of cases (Table 7). The core endemic area is centred on Switzerland, southern Germany, and eastern France.

In North Africa, there have been two reports of autochthonous AE from Tunisia and a further case from Morocco diagnosed by histological examination of the lesions [41], [42]. However the parasite has never been recorded in animals from Africa and in the absence of molecular confirmation there is insufficient evidence to confirm any part of North Africa is presently endemic for *E. multilocularis*.

AE is a serious disease and, although the prognosis is reasonably good when treatment is available [3], the prognosis is equally bleak in the absence of treatment. The overwhelming number of cases comes from an area of rural China which forms part of the Tibetan plateau. This population is remote and with few financial resources with an estimated annual income per head of less than US$500 [43]. Therefore it is reasonable to assume that most of these cases will be fatal and hence, the annual mortality due to AE is similar to the incidence. This is also likely to be true of most other cases outside of Europe. An annual mortality due to AE of approximately 18,000 is greater than one tenth of the total mortality of 177,000 as a result of the 10 diseases of the neglected tropical disease cluster (trypanosomiasis, Chagas disease, schistosomiasis, leishmaniasis, lymphatic filariasis, onchocerciasis, intestinal nematode infections, Japanese encephalitis, dengue, and leprosy ) [44]. The disease burden can also be compared to that of rabies. Annual AE mortality is approximately one third of that due to rabies which has been estimated at approximately 55,000 [45], [46]. Nearly all is from Africa and Asia. Unlike rabies, there is also no vaccine for canid echinococcosis and control requires repeated treatment of foxes or dogs with praziquantel. Thus, although AE is rare on a global scale it has a high burden in some highly endemic communities in China where it is likely to be one of the leading causes of death.

Likewise the global burden of disease, in terms of DALYs, is high. This is again due to the very high fatality rate resulting in a large number of YLLs but additionally due to the expected high disability weight that individuals have during the course of the disease. Diseases with similar magnitudes of DALYs include neglected tropical diseases such as onchocerciasis, and Chagas disease [47]. An initial estimate of the global burden of cystic echinococcosis was approximately 1 million DALYs [22]. However, this is likely to be an underestimate [48] and a re-evaluation of the global burden of CE is ongoing.

Control of this disease depends on the risk factors for transmission. The wild life cycle can be disturbed through the treatment of foxes with praziquantel impregnated baits [10]. However, the feasibility of applying such control measures over large parts of the Tibetan plateau, where the main burden of AE is found, would be questionable. Dog contact is a known risk factor for transmission to man [49] and dogs are highly susceptible to infection with this parasite [50]. However, it is not known if dogs participate in the cycle or are aberrant definitive hosts. If they are aberrant hosts, periodic treatment of dogs will not disturb the transmission cycle and will have much less effect on long term transmission rates to humans [43]. Other means of reducing the disease burden would be through better control of food or water supplies which may be contaminated with parasite eggs. Such control would be dependent on the attributable fraction of disease burden due to these transmission pathways and the cost effectiveness of such intervention strategies.
